# Analysis of *C9orf72* repeat length in progressive supranuclear palsy, corticobasal syndrome, corticobasal degeneration, and atypical parkinsonism

**DOI:** 10.1007/s00415-025-12990-9

**Published:** 2025-03-26

**Authors:** David P. Vaughan, Raquel Real, Marte Theilmann Jensen, Riona G. Fumi, Megan Hodgson, Edwin Jabbari, Danielle Lux, Lesley Wu, PROSPECT consortium, MD-GAP, Thomas T. Warner, Zane Jaunmuktane, Tamas Revesz, James B. Rowe, Jonathan Rohrer, Huw R. Morris

**Affiliations:** 1 Department of Clinical and Movement Neurosciences, UCL Queen Square Institute of Neurology, University College London, London, UK; 2 Movement Disorders Centre, UCL Queen Square Institute of Neurology, London, UK; 3 Queen Square Brain Bank, Reta Lila Weston Institute of Neurological Studies, UCL Queen Square Institute of Neurology, London, UK; 4 Department of Neurodegenerative Disease, Queen Square Institute of Neurology, University College London, London, UK; 5 Department of Clinical Neurosciences, Cambridge University Hospitals NHS Trust, and MRC Cognition and Brain Sciences Unit, University of Cambridge, Cambridge, UK; 6 Dementia Research Centre, UCL Queen Square Institute of Neurology, London, UK

**Keywords:** Parkinsonism, Genetics, *C9orf72*, Progressive supranuclear palsy, Corticobasal degeneration

## Abstract

**Background:**

Pathogenic hexanucleotide repeat expansions in *C9orf72* are the commonest genetic cause of frontotemporal dementia and/or amyotrophic lateral sclerosis. There is growing interest in intermediate repeat expansions in *C9orf72* and their relationship to a wide range of neurological presentations, including Alzheimer’s disease, Parkinson’s disease, progressive supranuclear palsy, corticobasal degeneration, and corticobasal syndromes.

**Aims:**

To assess the prevalence of intermediate *C9orf72* repeat expansions in a large cohort of prospectively-recruited patients clinically diagnosed with progressive supranuclear palsy (PSP), corticobasal syndrome (CBS), and atypical parkinsonism (APS), compared with healthy controls. We also sought to replicate the association between C9orf72 repeat length and CBD in neuropathologically confirmed cases.

**Methods:**

626 cases, including PSP (*n* = 366), CBS (*n* = 130), and APS (*n* = 53) from the PROSPECT study, and 77 cases with pathologically confirmed CBD were screened for intermediate repeat expansions in *C9orf72* using repeat-primed PCR. These were compared to controls from the PROSPECT-M-UK study and from the 1958 Birth Cohort.

**Results:**

There was no difference in the mean or largest allele size in any affected patient group compared with controls. A higher proportion of our affected cohort had large *C9orf72* repeat expansions compared to controls, but there was no difference when comparing the frequency of intermediate expansions between affected patients and controls. There was no relationship between repeat length and age at onset, level of disability, or survival.

**Conclusions:**

Intermediate expansions in *C9orf72* do not appear to be a genetic risk factor for PSP, CBS, CBD, or atypical parkinsonism. They are not associated with age at onset, disability, or survival in our study.

**Supplementary Information:**

The online version contains supplementary material available at 10.1007/s00415-025-12990-9.

## Introduction

Progressive supranuclear palsy (PSP), corticobasal degeneration (CBD), and multiple systems atrophy (MSA) are usually sporadic conditions. However, there is increasing recognition of the role of frontotemporal dementia (FTD)-related genes in these diseases. Mutations in *MAPT* have been reported in patients with a PSP phenotype [1] and rarely in those with CBS and CBD [2, 3]. Similarly, parkinsonism has been reported in about 40% of patients with *GRN* mutations [4]. Our group has previously reported IVS10 + 16 and L284R *MAPT* mutations in patients presenting with PSP syndromes and a *TBK1* mutation in a patient with corticobasal syndrome [5–7].

Large hexanucleotide (GGGCC) repeat expansions in *C9orf72* have been identified as the most common monogenic cause of frontotemporal dementia (FTD) and amyotrophic lateral sclerosis (ALS) [8]. The exact mechanism through which this mutation causes disease is yet to be determined, with both toxic gain of function and loss of function being suggested [9]. While the function of the C9orf72 protein is also poorly understood, several studies have shown that it plays a role in vesicle trafficking [10–12].

Pathologically, patients with pathogenic *C9orf72* repeat expansions have cytoplasmic accumulation of TAR DNA-binding protein 43 (TDP-43), representing frontotemporal lobar degeneration (FTLD-TDP pathology). This pathological entity is characterized by TDP-43 positive cytoplasmic inclusions and neurites, seen in the frontotemporal neocortex and dentate granule cells of the hippocampus. Lentiform neuronal intranuclear inclusions can also be seen, though more frequently in familial than sporadic cases. Glial TDP-43 pathology is seen in the subcortical white matter in all cases and in several subcortical locations [13].

In individuals without neurological disease, the *C9orf72* hexanucleotide repeat normally comprises 2–8 repeat units [14]. However, one UK study found very large *C9Orf72* expansions in 1 out of 700 individuals in the general population, indicating incomplete penetrance and potentially other factors influencing gene expression [15]. In contrast, the large expansions are highly penetrant in families with FTD or ALS and the expansions typically have hundreds to thousands of repeat units [16, 17]. However, putative pathogenic expansions as short as 22–24 repeats have been reported [18]. While the lower limit for pathogenic allele lengths is subject to much debate, 30 repeats is a widely accepted cut-off [19].

In a study of pathologically confirmed cases of CBD, Cali and colleagues screened 354 cases for intermediate expansions (defined as alleles between 17 and 30 units). Intermediate expansions were found in 3.7% of autopsy-confirmed CBD cases, compared to only 0.52% of controls, with an odds ratio (OR) of 3.6 (*p* = 0.00024). Furthermore, post hoc analysis found an association between repeat expansions as low as 10 repeats and CBD, with an OR of 1.26 (*p* = 0.026). There were no expansions larger than 29 repeats in this cohort. Interestingly, while large repeat expansions in *C9orf72* have been shown to decrease expression of the gene, they found increased expression in the cerebellar tissue of patients with > 17 repeats. There was also absence of pathologic RNA foci or dipeptide repeat protein aggregates [20]. This study suggests that intermediate repeat expansions in *C9orf72* may increase risk for CBD and that the pathogenesis in intermediate expansions may be different from that in large expansions. It also raises the possibility of treating CBD by targeting *C9orf72* expression. Other studies have shown that *C9orf72* may influence autophagy pathways leading to neurodegeneration, thus repeat expansions could feasibly contribute to other conditions including PSP and CBD [21].

Clinical trials of agents targeting *C9orf72* in ALS have already begun [22, 23]. If this expansion is shown to contribute to pathogenesis in other conditions, medications with proven benefit could be used to treat them too. For this reason, in this study, we evaluated a large cohort of patients with diagnoses of PSP, CBS, and indeterminate APS for intermediate expansions in *C9orf72* to investigate if they are associated with disease risk and if they influence clinical phenotypes, such as age of disease onset, progression to disability, or survival time after symptom onset. We also screened a large group of pathologically confirmed corticobasal degeneration cases from UK and Victoria brain banks.

## Methods

### Participants

Following informed consent, we extracted DNA from blood (*n* = 542) or saliva (*n* = 12) from patients in the PROSPECT-M-UK study. The PROSPECT-M-UK study cohort is a UK-wide study of PSP, CBS, MSA, and indeterminate APS [24]. We identified participants with a diagnosis of PSP, CBS, CBD, or indeterminate APS based on pathological examination, if available, or best clinical diagnosis.

We identified cases from brain banks with a primary pathological diagnosis of CBD. DNA was extracted from brain tissue. CBD was defined following standard pathological criteria. Cases were excluded if they had been included in the previously published study [20].

We identified 86 participants unaffected by neurological disease from the PROSPECT-M-UK study, who had DNA extracted from blood, and also used data from previously published cohort of healthy controls (1958 birth cohort) [15].

The PROSPECT-M-UK study was approved by the National Research Ethics Service Committee London–Queen Square and complies with the ethical standards of the Declaration of Helsinki.

### Measuring *C9orf72* repeat length

We measured C9orf72 repeat length using the DeJesus-Hernandez repeat-primed PCR protocol [25], which can accurately size repeat expansions up to approximately 50 repeats. Briefly, we combined Amplitaq Gold 360 master Mix, Betaine, and a mix of 3 *C9orf72* hexanucleotide PCR primers with 1 uL of DNA at a concentration of 100 ng/uL in each well of a 96-well plate and ran this on a PCR cycle. We prepared an electrophoresis gel from HiFi Formamide and GeneScan 500 LIZ dye size standard and added the PCR product to this in another 96-well plate. This was heated to 95 °C for 3 min and then immediately cooled to 4 °C and before being read by the Applied Biosystems (ABI) 3130xl Genetic Analyzer.

### Determining repeat size

We determined allele length using Geneious Prime software (version 2024.0.5, Dotmatics). Allele length in base pairs was converted to repeat size using the following formula to remove the sequence in the PCR amplicon that flanks the repeat and add a 2 base pair correction factor: (Allele size - 118.7)/5.8. Samples with homozygous alleles of 2–3 repeats in the DeJesus protocol have an appearance that leads to incorrect calling by the software at repeat size 8. There is characteristically one small peak at the 2–3 repeat locus and a second small peak at the 8 repeat locus without a saw-tooth appearance, which is a recognized artifact (Supplementary Fig. 1A). True 8 repeat alleles have high signal intensity and a characteristic saw-tooth appearance (Supplementary Fig. 1B). We visually inspected the electropherograms in Geneious Prime and manually corrected the incorrectly called peak at repeat size 8 in cases of homozygous 2–3 repeats before statistical analysis.

### Statistical analysis

We performed all statistical analysis using R. We compared the mean age of onset (age at sampling for controls) and sex, self-reported ethnicity and family history of neurodegenerative disease between PSP, CBS, APS, and CBD and unaffected controls using Kruskal–Wallis rank sum test and Chi-squared test, respectively. Analysis of repeat sizes was performed on an allelic basis (i.e., 2 alleles for each individual). We defined the proportion of cases and controls with pathogenic *C9orf72* expansions (> 30 repeats). We compared frequency of intermediate repeat alleles (≥ 17 repeats) for pathologically confirmed CBD, and clinically diagnosed PSP, CBS, and APS using Fisher’s exact test and repeated this for each cut-off of repeat size between 4 and 29. We then investigated the effect of repeat size on age at onset, disability at baseline (determined by the score on the Schwab and England scale) using linear regression with sex, and sex and disease duration at time of assessment as covariates, respectively. Finally, we examined the effect of repeat size on survival using the Cox proportional hazard model in a subgroup of affected patients where mortality data were available at each cut-off for repeat size between 3 and 30.

## Results

We screened 77 patients with pathologically confirmed CBD and 549 patients with clinically diagnosed PSP (*n* = 366), CBS (*n* = 130), and APS (*n* = 53) for intermediate repeat expansions in *C9orf72*. The demographic characteristics of participants are shown in Table 1. A higher proportion of the control subjects and CBS patients were female (59.3% and 58.5%, respectively, *p* = 0.034). There was no difference in age at onset between the patient groups and age at blood sampling for controls. The highest rate of family history of neurodegenerative disease was found in the CBS group (85.7% in CBS vs 25.8% in CBD, *p* < 0.001). There was a higher rate of large *C9orf72* repeat expansions in our affected patients compared to healthy controls (0.3% vs 0.1%, OR = 4.41, *p* = 0.023).

**Table 1:** Demographic features of cases and controls with mean C9orf72 repeat length by group

	Diagnoses					*p* value
	Clinical			Pathological	Control	
	PSP	CBS	APS	CBD		
*n*	366	130	53	77	86	
Age at onset/sampling mean (SD)	66.6 (7.0)	65.4 (7.8)	67.1 (8.7)	65.6 (7.2)	67.4 (7.5)	0.274
Female *n* (%)	172 (47.0%)	76 (58.5%)	25 (47.2%)	32 (41.6%)	51 (59.3%)	0.034^a^
Family history (*n *= 134)						
Neurodegenerative disease *n* (%)	45 (61.6)	18 (85.7)	4 (44.4)	8 (25.8)		< 0.001^b^
Dementia *n* (%)	20 (27.4)	11 (52.4)	2 (22.2)	2 (6.5)		0.003^c^
Parkinsonism *n* (%)	22 (30.1)	8 (38.1)	0 (0)	1 (3.2)		0.003^d^
ALS/MND *n* (%)	3 (4.1)	0 (0)	0 (0)	1 (3.2)		0.742
Other neurodegenerative disease *n* (%)	2 (2.7)	0 (0)	2 (22.2)	0 (0)		0.004
Not otherwise specified *n* (%)	0 (0)	0 (0)	0 (0)	4 (12.9)		0.003
Self-reported ancestry (*n* = 559)						
EUR *n* (%)	348 (95.9%)	125 (96.2%)	51 (96.2%)	11 (84.6%)	85 (98.8%)	0.001^e^
AFR *n* (%)	3 (0.8%)	0 (0%)	1 (1.9%)	1 (7.7%)	0 (0%)	
EAS *n* (%)	0 (0%)	1 (0.8%)	0 (0%)	1 (7.7%)	0 (0%)	
SAS *n* (%)	11 (3%)	4 (3.1%)	1 (1.9%)	0 (0%)	1 (1.2%)	
MDE *n* (%)	1 (0.3%)	0 (0%)	0 (0%)	0 (0%)	0 (0%)	
*C9orf72* repeat length mean (SD)	4.3 (4.3)	4.0 (3.2)	3.7 (2.8)	4.1 (4.6)	3.9 (2.8)	0.563
Proportion of cases < 4 repeats	61.7%	64.6%	66.0%	66.2%	61.6%	0.658

*EUR* European, *AFR* African/African-Caribbean, *EAS* East Asian, *SAS* South Asian, *MDE* Middle Eastern

Note UK 2021 national average EUR 94% in people over 65 years (Office for National Statistics)

^a^*p* value significant for CBD vs control, PSP, vs CBS, CBS vs CBD

^b^*p* value significant for PSP vs CBD, CBS, vs CBD

^c^*p* value significant for PSP vs CBD, CBS vs CBD

^d^*p* value significant for PSP vs CBD, CBS vs CBD

^e^*p* value significant for CBD vs Control, PSP vs CBD, CBS vs CBD

We found alleles ≥ 30 repeats and ≥ 17 repeats in 0.3% (*n* = 4) and 0.6% (*n* = 7) of the affected patients alleles, respectively. The largest allele in our cohort was 65 repeats, found in a male presenting with progressive gait difficulties including falls and gait freezing from the age of 70. Examination revealed reduced blinking with frontalis overactivity, delay in initiation of saccades but normal range and velocities, symmetric limb rigidity, and marked gait freezing. He progressed to using a wheelchair within 18 months of onset and he was not responsive to levodopa. MRI head showed disproportionate midbrain atrophy. His phenotype was most in keeping with PSP-progressive gait freezing (PSP-PGF). He passed away after a disease duration of 6 years, which is unusually fast for the PSP-PGF phenotype. A male patient with 47 repeats was diagnosed with FTD with symptom onset at age 60 and a disease duration of 10 years. The primary pathology on post-mortem was CBD.

A female patient with 45 repeats had onset of balance problems and speech apraxia at age 65. She was diagnosed with PSP. MRI brain showed symmetrical cortical atrophy in a fronto-parieto-temporal distribution. She passed away at the age of 77 after a disease duration of 12 years. Finally, a male patient with 30 repeats experienced onset at age 62 of an asymmetric syndrome involving rest tremor and balance impairment, developing a vertical supranuclear gaze palsy, dysarthria, and urinary incontinence after about 9 years. MRI brain showed disproportionate midbrain atrophy. He was diagnosed initially with Parkinson’s disease, but this was later revised to PSP parkinsonism (PSP-P). However, with the supranuclear gaze palsy, he probably met movement disorder criteria for PSP-RS. He passed away at age 78 after a total disease duration of 16 years. Neuropathological examination was not performed for any of the PSP patients and none of them had known family history of neurological disease. These cases are summarized in Table 2.

**Table 2:** Summary of patients with ≥ 30 repeats

	*C9orf72* allele sizes	Sex	AAO	Clinical diagnosis	Pathological diagnosis	Disease duration (years)	Family history
Patient 1	2, 65	Male	70	PSP-PGF	NA	6	No
Patient 2	2, 47	Male	60	FTD	CBD	10	Unknown
Patient 3	5, 45	Female	65	PSP	NA	12	No
Patient 4	2, 30	Male	62	PSP-P	NA	16	No

PSP-PGF PSP with Progressive Gait Freezing, *PSP-P PSP* parkinsonism, *AAO* Age at onset

**Fig. 1:**
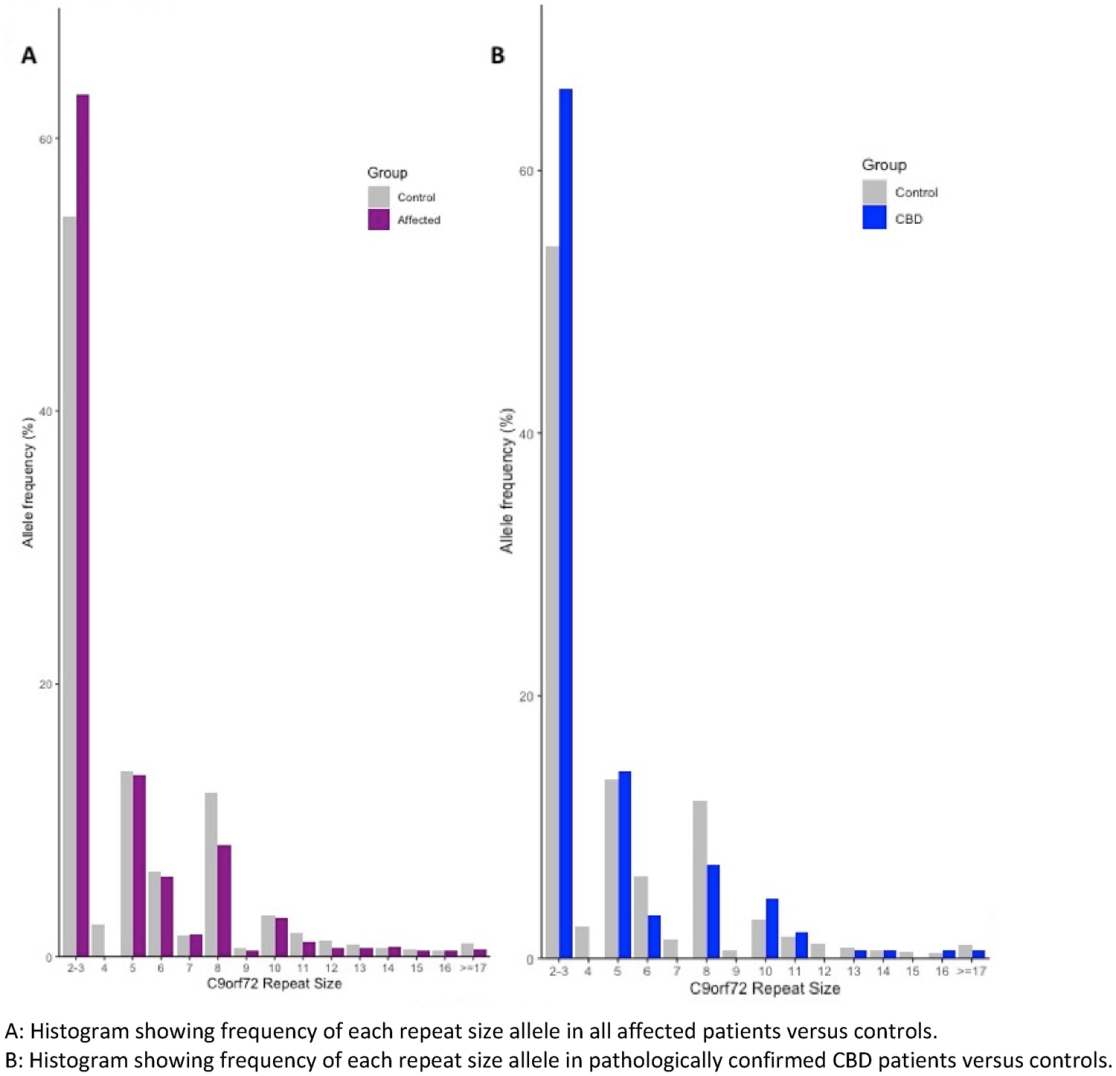
Allele frequencies of C9orf72 repeat sizes in cases and 1958 birth cohort controls. **A** Histogram showing frequency of each repeat size allele in all affected patients versus controls. **B** Histogram showing frequency of each repeat size allele in pathologically confirmed CBD patients versus controls

**Fig. 2:**
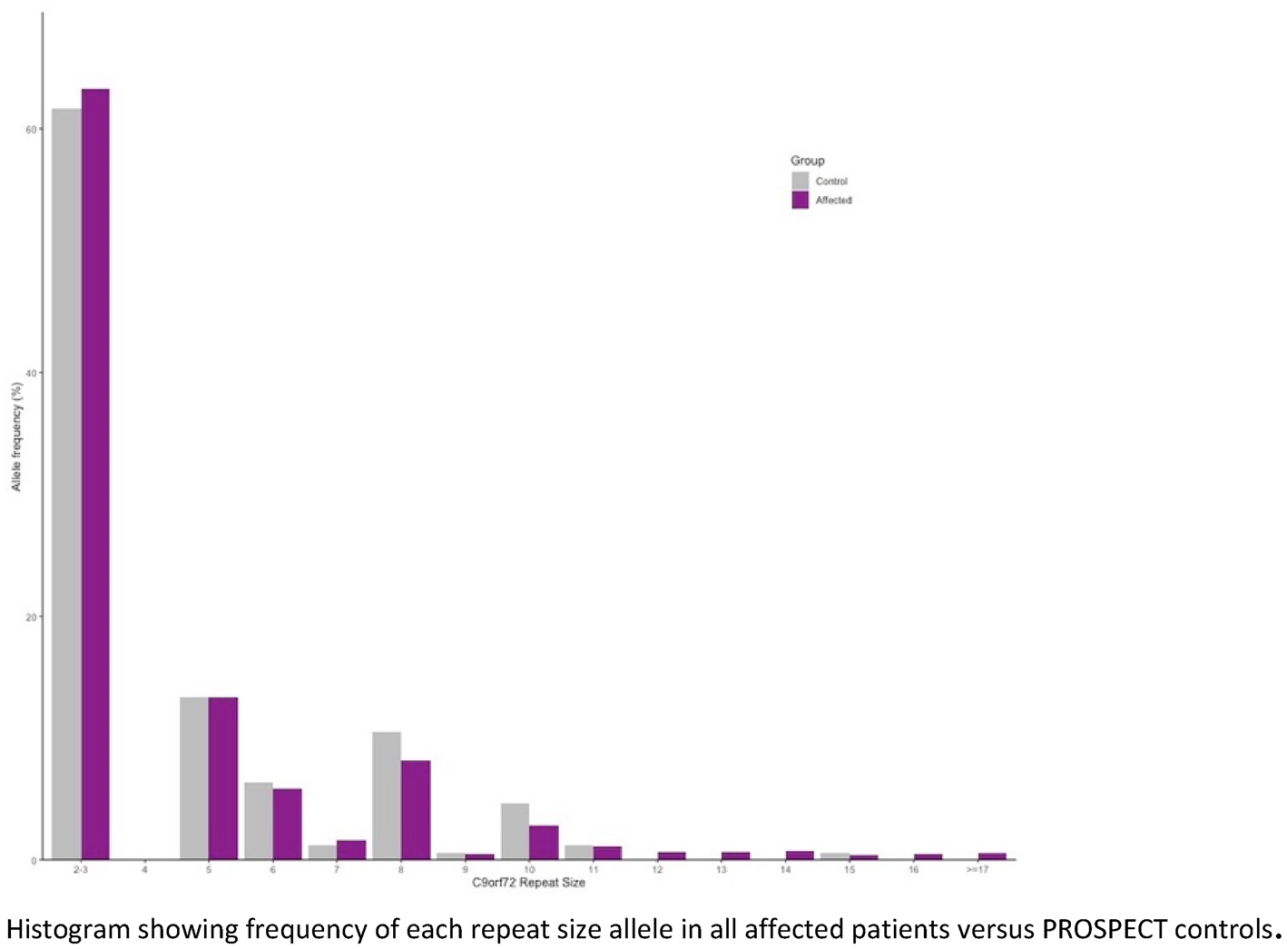
Allele frequencies of C9orf72 repeat sizes in cases and 1958 birth cohort controls. Histogram showing frequency of each repeat size allele in all affected patients versus PROSPECT controls

The healthy control cohort had a higher proportion of intermediate alleles compared to the affected patient cohort (0.9% vs 0.2%, *p* = 0.007). The affected patient cohort had a *higher* proportion of “normal” alleles (2–3 repeats) and a lower proportion of 8 repeat alleles compared to the 1958 birth cohort (Fig. 1, Supplementary Table 1). For all groups, there was no difference in mean allele size and no difference in the proportion of alleles with intermediate expansions (≥ 17 repeats), or any cut-off of allele size between 9 and 29 compared with controls, after Bonferroni correction for multiple comparisons (Table 3 and Supplementary Table 2). At a cut-off of ≥ 8 repeats, larger alleles were more common in the control group compared to the affected patient group, even after Bonferroni correction (OR 0.67, *p* value < 0.001) (Supplementary Table 2). This was likely due to the lower proportion of “normal” alleles in the control group. As described above, homozygous 2–3 repeat alleles can be incorrectly called as having one normal and one 8 repeat allele. In our data, we manually corrected this calling error before statistical analysis. To substantiate this claim, we performed a separate analysis between our patients and the healthy controls from PROSPECT-M-UK. A difference between the proportion of “normal” and eight repeat alleles was not seen (Fig. 2, Table 3).

**Table 3:** Large (≥ 30) C9orf72 repeat expansions and repeat expansions ≥ 17 in patients compared with controls using Fisher’s exact test

Diagnosis	Total alleles	≥ 30 Repeats	< 30 Repeats	% ≥ 30	Odds ratio	95% CI	*p* value
Controls	15,154	11	15,143	0.1	**–**	**–**	**–**
All affected	1252	4	1248	0.3	4.41	1.02–14.92	0.023
PSP	732	3	719	0.4	5.66	1.01–21.5	0.024
CBS	260	0	260	0	**–**	**–**	**–**
APS	106	0	106	0	Inf	0.02–Inf	1
CBD	154	1	153	0.6	8.99	0.21–62.61	0.114

Diagnosis	Total alleles	≥ 17 Repeats	< 17 Repeats	% Intermediate	Odds ratio	95% CI	*p* value^a^
Controls	15,154	153	15,001	1.0			
All affected	1252	7	1245	0.6	0.55	0.42–2.40	0.134
PSP	732	5	727	0.7	0.67	0.22–1.62	0.564
CBS	260	1	259	0.4	0.38	0.01–2.16	0.526
APS	106	0	106	0.0	0.00	0.00–3.51	0.63
CBD	154	1	153	0.6	0.64	0.02–3.68	1

^a^ Bonferroni correction for multiple comparisons applied for 25 intermediate allele cut-offs, giving significance level *p* = 0.002

We found no relationship between repeat length and age at symptom onset or disability as measured on the Schwab and England scale. Unit increases in repeat size are statistically associated with a very marginal increase the risk of the death, but, in practice, there is no effect as the hazard ratio is close to 1 (HR 1.02, *p* = 0.024) (Table 4). We performed Cox proportional hazard analysis with repeat size as a binary variable at every cut-off between 3 and 30 alleles. There was no significant effect of repeat length on survival after Bonferroni correction (Supplementary Table 3).

**Table 4:** Linear regression/Cox proportional hazard modeling of repeat length as a determinant of age at onset, Schwab and England score at baseline and survival with covariate as indicated

Diagnosis	All		PSP		CBS		APS		CBD	
	HR (95% CI)	*p* value	HR (95% CI)	*p* value	HR (95% CI)	*p* value	HR (95% CI)	*p* value	HR (95% CI)	*p* value
Age at onset beta	− 0.01 (− 0.14 to 0.13)	0.93	0.01 (− 0.13 to 0.15)	0.858	0.04 (− 0.33 to 0.41)	0.837	− 0.44 (− 1.15 to 0.27)	0.218	0 (− 0.3 to 0.3)	0.997
SEADL beta	0.53 (− 0.26 to 1.33)	0.187	0.97 (-− 0.1 to 2.05)	0.076	0.47 (− 0.98 to 1.92)	0.52	− 1.34 (− 3.76 to 1.07)	0.261	–	–
Survival HR, continuous variable	1.02 (0.98 to 1.00)	0.024	0.99 (0.96 to 1.02)	0.989	0.92 (0.85 to 1.0)	0.040	0.97 (0.85 to 1.11)	0.689	1.02 (0.95 to 1.11)	0.565

*SEADL* Schwab and England Activities of daily living scale, *HR* Hazard ratio, *CI* confidence interval

Linear regression applied for age at onset (sex as covariate) and SEADL (sex and disease duration at assessment as covariates)

Cox proportional hazard regression for survival (sex and age at death or censoring as covariates)

## Discussion

In this study, we screened for intermediate expansions in *C9orf72* in 549 cases of PSP, CBS and indeterminate APS, as well as 77 cases with pathologically confirmed CBD, and compared this to a large healthy control cohort. We did not confirm the hypothesis that such intermediate expansions in *C9orf72* are a genetic risk factor for CBD [20]. We found no relationship between *C9orf72* repeat length and age at disease onset or physical independence at baseline assessment. The cut-off for intermediate repeat expansions is the subject of much debate. Previous studies have quoted arbitrary cut-offs of 17 repeats, 20 repeats, and 22 repeats [20, 26, 27]. To address this issue, we performed our analysis at every cut-off between 9 and 29 repeats and found no correlation at any of these.

We found 4 cases with one allele ≥ 30 repeats. Three had features of PSP and one had features of FTD, with pathologically confirmed CBD. None had features of ALS or FTD or family history of neurological disease. Neuropathological examination was not available for the PSP cases to confirm if they had PSP (4-repeat tau) or TDP-43 pathology. It is interesting that only one of these patients had clinical features of FTD but without typical pathology associated with *C9orf72* repeat expansions. One explanation for this is that while cases of FTD/ALS with repeats in this range have been reported, most have much larger repeat sizes. Furthermore, as discussed below, promoter hypermethylation can influence gene expression in *C9orf72* repeat expansions, so potentially the expansion has been silenced in this way.

Our clinical cohort had a higher proportion of 2–3 repeat alleles and a lower proportion of 8 repeat alleles than the healthy control population, which is likely due to a difference in the methodology in repeat size calling. This accounts for the differences seen between allele sizes in patients and controls at all cut-offs below 8 repeats.

We saw 3 allele sizes that occur more often in all groups; 2–3 repeats, 5 repeats, and 8 repeats (Supplementary Table 1). This is consistent with reports from previous studies of multiple haplotypes in the gene [19, 25, 28]. This suggests that the “risk” haplotype, which has an average of 8 repeats, is more unstable and prone to slippage, leading to pathological expansions in seemingly sporadic cases of ALS and FTD.

While pathogenesis in patients with large *C9orf72* repeat expansions is not well understood, proposed disease mechanisms include haploinsufficiency leading to excitotoxicity, which is linked to glutamate receptor accumulation, and impaired clearance of neurotoxic dipeptide repeat proteins (loss of function). Alternatively, pathological and induced pluripotent stem cell (iPSC) studies have identified RNA foci and dipeptide repeat proteins transcribed and translated from the repeat expansion which interact with RNA-binding proteins and induce neurodegeneration (toxic gain of function). It is possible that there is interplay between loss of function and gain of function mechanisms, with one driving production of neurotoxic inclusions and one interfering with the brain’s ability to clear them, each contributing to the observed neurodegeneration [19, 21, 25, 29, 30].

Significant phenotypic variation has been reported in patients with a pathogenic *C9orf72* expansion. Approximately half of patients with *C9orf72* expansions develop parkinsonism [31, 32]. Furthermore, some studies, including a meta-analysis, have suggested that intermediate expansions may be a risk factor in clinically diagnosed Parkinson’s disease (PD) [33, 34]. However, this was not confirmed on a subsequent study of 488 cases with pathologically confirmed PD [35]. Additionally, several studies with relatively small patient numbers have shown an association between large and intermediate expansions and clinically diagnosed atypical parkinsonian syndromes [26, 27]. Again, screening of pathologically confirmed cases of PSP, CBD, and MSA did not have any expansions larger than 22 repeat units [27].

Intermediate tandem repeats that cause a different phenotype to the “full” expansion have previously been reported with *ATXN2*. Polyglutamine expansions of ≥ 34 repeats in this gene are known to cause spinocerebellar ataxia type 2 (SCA2) [36–39]. However, intermediate expansions in the range of 27–31 were found to increase toxicity of TDP-43 and thus cause an ALS phenotype [40]. These intermediate *ATXN2* expansions in ALS have been reported in multiple populations [41–46]. Furthermore, longer *ATXN2* repeat length leads to shorter survival in ALS [42].

Several studies have attempted to investigate the relationship between repeat length and other aspects of clinical phenotype in *C9orf72*. A positive correlation between repeat length and age of onset has been reported by Van Blitterwijk and colleagues who found this relationship by measuring repeat length in the frontal lobes of patients with FTD/ALS [47]. However, Nordin et al*.* could not confirm this but did find a positive correlation between repeat length in the parietal lobe and cerebellum in ALS patients only [48]. Conversely, another group reported an inverse relationship, with longer repeat length being associated with earlier age of onset [49]. Other correlations with repeat length have been suggested. For example, Van Blitterswijk and colleagues also found a correlation between increased length and poorer survival after disease onset [47]. Our study did not show any effect of *C9orf72* repeat size on survival in patients with PSP, CBS, CBD, or APS.

There is significant somatic instability, with variation in repeat length between different tissues and brain areas. However, less variability and increased correlation with phenotype have been reported in the cerebellum [47–49]. The presence of somatic expansions was also confirmed by Fratta and colleagues [50]. They demonstrated repeat lengths between 900 and 3000 + units in CNS tissues of a patient with approximately 90 repeat units in blood. Furthermore, other non-CNS tissues were also found to have this smaller repeat, suggesting that the expansion in repeat size occurred in the developmental lineage after the differentiation between central and peripheral nervous system. However, they screened 8 samples with intermediate repeat expansions of 20–27 units for somatic expansions in the CNS and did not find any. Another group has also shown variability in repeat length measured in peripheral blood, depending on the age at which the blood is collected, possibly explained by the regenerating nature of blood [47]. DNA from patients in our PROSPECT-M-UK cohort was mostly extracted from peripheral blood, but 71/77 (92.2%) of our CBD cases had DNA extracted from brain tissue. The specific brain region was known in 19/71 brain bank cases; 13 were from cerebellum, and 6 were from frontal cortex. Use of brain-derived DNA if available in our clinical cohort may have increased detection of repeat expansions. However, we note that only one expansion ≥ 17 repeats was found in the brain-derived DNA of our pathological CBD cohort.

Beck and colleagues reported that 1 in 700 healthy individuals in the UK population carry large *C9orf72* repeat expansions [15]. This may explain why there are conflicting results from investigations into the relationship between repeat length and phenotype. It suggests that there are other factors contributing to pathogenesis and phenotype. One of these factors is methylation. As in diseases caused by trinucleotide repeats, promoter hypermethylation of *C9orf72* appears to modify gene expression. Multiple studies have suggested that 5’ promoter hypermethylation results in epigenetic silencing of *C9orf72* repeat expansions and may therefore be protective [51–53]. Russ et al*.* examined 118 *C9orf72* repeat expansion carriers with FTD or ALS. They found no difference in the degree of promoter hypermethylation between patients in the 2 disease groups, nor did it affect age at onset or disease duration. However, in the FTD cohort, there was a significant association between methylation, measured in peripheral blood, and age at death, with increases in methylation correlating with later age at death (i.e., increased survival). Furthermore, they showed that promoter hypermethylation is stable across several neuronal and non-neuronal tissues and that it does not vary with age at collection of peripheral blood, unlike *C9orf72* repeat length [54]. Therefore, methylation may be a useful predictor of clinical phenotype in addition to repeat length, and it may have utility as a potential biomarker.

While this study suggests that repeat expansions in *C9orf72* are not a genetic risk factor for PSP, CBS, or APS, there is evidence to suggest a genetic basis in familial forms of these conditions, particularly tauopathies. These familial forms can help understand pathogenesis in sporadic cases and lead to the development of novel treatments. For example, mutations in *MAPT* are a widely reported cause of tauopathy, with varying phenotypic presentations including FTD, PSP, and CBS. Effects of these mutations include altered alternative tau splicing, leading to an altered 3R/4R tau ratio; aberrant tau phosphorylation; impaired microtubule assembly; and promotion of tau aggregation [4, 55–60]. Other genes have also been implicated, such as *LRRK2*, which is an established cause of familial Parkinson’s disease, but may also contribute to other forms of neurodegeneration through overstabilisation of the actin cytoskeleton [61]. Several other mechanisms contribute to neurodegeneration, including acceleration of protein aggregation and disruption of the normal protein clearance pathways, and represent potential treatment targets [62].

This is the largest study to date on intermediate repeat expansions in *C9orf72* in clinically diagnosed patients with PSP, CBS, and indeterminate atypical parkinsonism. As most of the data were collected prospectively, we were able to assess the effect of intermediate expansions on survival. The main limitation of this study is that we only included 77 cases of pathologically confirmed CBD, whereas the previous study had 354. None of the patients included in that study were included in the present study. Similar to the previous study, we also used repeat size data from a previously published cohort of healthy controls. While this greatly increased the number of controls for analysis, we did not have individual level data on age and sex. We recognize that generational effects may reduce power as the control cohort was born a mean of 12 years later than our cases. However, work by Beck and colleagues [15] with three different control cohorts did not show generational differences in allele distribution. There may be an effect of age at sampling in case–control comparisons, but this is unlikely to be relevant to our analysis as our cases were 10–12 years older at blood draw and the previous work has shown evidence for an increase in tandem repeat length with age [47].

In conclusion, we did not find any evidence to support that intermediate repeat expansions in *C9orf72* are a genetic risk factor for PSP, CBS, or APS, or that *C9orf72* repeat size has a modifying effect on age at onset, disease severity at baseline assessment, or survival in this cohort. The findings of Cali and colleagues were not replicated. Further studies in larger pathologically confirmed cohorts are needed, as well as further work into epigenetic factors influencing *C9orf72* gene expression.

## Supplementary Information

Below is the link to the electronic supplementary material.

Supplementary file1 (DOCX 37 kb)

Supplementary file2 (DOCX 17 kb)

Supplementary file3 (DOCX 51 kb)

Supplementary file4 (DOCX 21 kb)

Supplementary file5 (PDF 90 kb)

## Data Availability

All data used in this study can be accessed by request to the corresponding author.
