# Supplementary material for: Analysis of C9orf72 repeat length in progressive supranuclear palsy, corticobasal syndrome, corticobasal degeneration, and atypical parkinsonism
Source: J Neurol. 2025 Mar 26;272(4):293. doi: 10.1007/s00415-025-12990-9 (PMC11947049; doi:10.1007/s00415-025-12990-9)
Supplement: Supplementary file 1 — Supplementary file1 (DOCX 37 kb) [file 415_2025_12990_MOESM1_ESM.docx]

**Analysis of *C9orf72* repeat length in progressive supranuclear palsy, corticobasal syndrome, corticobasal degeneration and atypical parkinsonism**

**Journal of Neurology**

**Author List:** David P Vaughan (1,2), Raquel Real (1,2), Marte Theilmann Jensen (1,2), Riona G Fumi (1,2), Megan Hodgson (1,2), Edwin Jabbari (1,2), Danielle Lux (1,2), Lesley Wu (1,2), PROSPECT consortium, MD-GAP, Tom Warner (1,2), Zane Jaunmuktane (2,3), Tamas Revesz (3, 4), James B Rowe (4), Jonathan Rohrer (5), Huw R Morris (1,2)

**Corresponding author:** Huw Morris (h.morris@ucl.ac.uk)- Department of Clinical and Movement Neurosciences, UCL Queen Square Institute of Neurology, University College London, London, UK

**Supplementary Figure 1**

**Supplementary Figure 1: Heterozygous 2-3 repeat allele trace vs true 8 repeat allele trace**

*
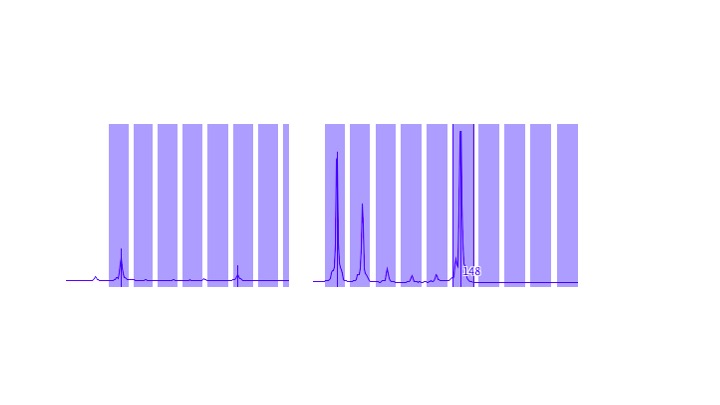
*
