# Supplementary material for: Analysis of C9orf72 repeat length in progressive supranuclear palsy, corticobasal syndrome, corticobasal degeneration, and atypical parkinsonism
Source: J Neurol. 2025 Mar 26;272(4):293. doi: 10.1007/s00415-025-12990-9 (PMC11947049; doi:10.1007/s00415-025-12990-9)
Supplement: Supplementary file 2 — Supplementary file2 (DOCX 17 kb) [file 415_2025_12990_MOESM2_ESM.docx]

**Analysis of *C9orf72* repeat length in progressive supranuclear palsy, corticobasal syndrome, corticobasal degeneration and atypical parkinsonism**

**Journal of Neurology**

**Author List:** David P Vaughan (1,2), Raquel Real (1,2), Marte Theilmann Jensen (1,2), Riona G Fumi (1,2), Megan Hodgson (1,2), Edwin Jabbari (1,2), Danielle Lux (1,2), Lesley Wu (1,2), PROSPECT consortium, MD-GAP, Tom Warner (1,2), Zane Jaunmuktane (2,3), Tamas Revesz (3, 4), James B Rowe (4), Jonathan Rohrer (5), Huw R Morris (1,2)

**Corresponding author:** Huw Morris (h.morris@ucl.ac.uk)- Department of Clinical and Movement Neurosciences, UCL Queen Square Institute of Neurology, University College London, London, UK

**Supplementary Table 1**

***Supplementary Table 1: Frequency of each C9orf72 allele size by diagnosis***

| **Repeat size** | **All Affected**  **(n = 1252)** | **PSP** | **CBS** | **APS** | **CBD** | **Controls** | **p-value**  **(all vs controls)^a^** |
| --- | --- | --- | --- | --- | --- | --- | --- |
| n alleles |  | 732 | 260 | 106 | 154 | 15326 |  |
| 2-3 | 792 (63.3%) | 452 (61.7%) | 168 (64.6%) | 70 (66.0%) | 102 (66.2%) | 9119 (55.0%) | <0.001 |
| 4 | 0 | 0 | 0 | 0 | 0 | 361 (2.2%) |  |
| 5 | 167 (13.3%) | 102 (13.9%) | 28 (10.8%) | 15 (14.2%) | 22 (14.3%) | 2257 (13.6%) |  |
| 6 | 73 (5.8%) | 43 (5.9%) | 17 (6.5%) | 8 (7.5%) | 5 (3.2%) | 1029 (6.2%) |  |
| 7 | 20 (1.6%) | 16 (2.2%) | 3 (1.2%) | 1 (0.9%) | 0 | 250 (1.5%) |  |
| 8 | 102 (8.1%) | 57 (7.8%) | 30 (11.5%) | 4 (3.8%) | 11 (7.1%) | 1939 (11.7%) |  |
| 9 | 6 (0.5%) | 4 (0.5%) | 2 (0.8%) | 0 | 0 | 96 (0.6%) |  |
| 10 | 35 (2.8%) | 20 (2.7%) | 3 (1.2%) | 5 (4.7%) | 7 (4.5%) | 497 (3.0%) |  |
| 11 | 14 (1.1%) | 9 (1.2%) | 2 (0.8%) | 1 (0.9%) | 3 (1.9%) | 277 (1.7%) |  |
| 12 | 8 (0.6%) | 4 (0.5%) | 4 (1.5%) | 0 | 0 | 190 (1.1%) |  |
| 13 | 8 (0.6%) | 6 (0.8%) | 0 | 1 (0.9%) | 1 (0.6%) | 139 (0.8%) |  |
| 14 | 9 (0.7%) | 6 (0.8%) | 2 (0.8%) | 1 (0.9%) | 1 (0.6%) | 103 (0.6%) |  |
| 15 | 5 (0.4%) | 4 (0.5% | 0 | 1 (0.9%) | 0 | 90(0.5%) |  |
| 16 | 6 (0.5%) | 4 (0.5%) | 1 (0.4%) | 0 | 1 (0.6%) | 71 (0.4%) |  |
| >=17 | 7 (0.6%) | 5 (0.7%) | 1 (0.4%) | 0 | 1 (0.6%) | 160 (1.0%) |  |

^a^ p-value from chi-squared test
