# Supplementary material for: Analysis of C9orf72 repeat length in progressive supranuclear palsy, corticobasal syndrome, corticobasal degeneration, and atypical parkinsonism
Source: J Neurol. 2025 Mar 26;272(4):293. doi: 10.1007/s00415-025-12990-9 (PMC11947049; doi:10.1007/s00415-025-12990-9)
Supplement: Supplementary file 3 — Supplementary file3 (DOCX 51 kb) [file 415_2025_12990_MOESM3_ESM.docx]

**Analysis of *C9orf72* repeat length in progressive supranuclear palsy, corticobasal syndrome, corticobasal degeneration and atypical parkinsonism**

**Journal of Neurology**

**Author List:** David P Vaughan (1,2), Raquel Real (1,2), Marte Theilmann Jensen (1,2), Riona G Fumi (1,2), Megan Hodgson (1,2), Edwin Jabbari (1,2), Danielle Lux (1,2), Lesley Wu (1,2), PROSPECT consortium, MD-GAP, Tom Warner (1,2), Zane Jaunmuktane (2,3), Tamas Revesz (3, 4), James B Rowe (4), Jonathan Rohrer (5), Huw R Morris (1,2)

**Corresponding author:** Huw Morris (h.morris@ucl.ac.uk)- Department of Clinical and Movement Neurosciences, UCL Queen Square Institute of Neurology, University College London, London, UK

**Supplementary Table 2**

***Supplementary Table 2: Intermediate C9orf72 repeat expansions in patients compared with controls using Fisher's exact test***

| **Diagnosis** | **Total Alleles** | **≥29 Repeats** | **<29 Repeats** | **% ≥29** | **Odds ratio** | **95% CI** | **p-value^a^** |
| --- | --- | --- | --- | --- | --- | --- | --- |
| **Controls** | 15154 | 13 | 15141 | 0.1 | - | - | - |
| **CBD** | 154 | 1 | 153 | 0.6 | 7.61 | 0.18 to 51.27 | 0.132 |
| **All affected** | 1252 | 5 | 1247 | 0.4 | 4.67 | 1.3 to 13.99 | 0.009 |
| **PSP** | 732 | 3 | 729 | 0.4 | 4.79 | 0.87 to 17.49 | 0.035 |
| **CBS** | 260 | 1 | 259 | 0.4 | 4.5 | 0.12 to 30.16 | 0.212 |
| **APS** | 106 | 0 | 106 | 0.0 | - | - | - |
|  |  | **≥27 Repeats** | **<27 Repeats** | **% ≥27 Repeats** | **Odds ratio** | **95% CI** | **p-value^a^** |
| **Controls** | 15154 | 16 | 15138 | 0.1 | - | - | - |
| **CBD** | 154 | 1 | 153 | 0.6 | 6.19 | 0.15 to 40.33 | 0.158 |
| **All affected** | 1252 | 5 | 1247 | 0.4 | 3.79 | 1.08 to 10.86 | 0.019 |
| **PSP** | 732 | 3 | 729 | 0.4 | 3.89 | 0.73 to 13.64 | 0.054 |
| **CBS** | 260 | 1 | 259 | 0.4 | 3.65 | 0.09 to 23.68 | 0.251 |
| **APS** | 106 | 0 | 106 | 0.0 | - | - | - |
|  |  | **≥26 Repeats** | **<26 Repeats** | **% ≥26 Repeats** | **Odds ratio** | **95% CI** | **p-value^a^** |
| **Controls** | 15154 | 17 | 15137 | 0.1 | - | - | - |
| **CBD** | 154 | 1 | 153 | 0.6 | 5.82 | 0.14 to 37.59 | 0.166 |
| **All affected** | 1252 | 5 | 1247 | 0.4 | 3.6 | 1.03 to 10.10 | 0.023 |
| **PSP** | 732 | 3 | 729 | 0.4 | 3.66 | 0.69 to 12.71 | 0.062 |
| **CBS** | 260 | 1 | 259 | 0.4 | 3.44 | 0.08 to 22.09 | 0.264 |
| **APS** | 106 | 0 | 106 | 0.0 | - | - | - |
|  |  | **≥25 Repeats** | **<25 Repeats** | **% ≥25 Repeats** | **Odds ratio** | **95% CI** | **p-value^a^** |
| **Controls** | 15154 | 22 | 15132 | 0.1 | - | - | - |
| **CBD** | 154 | 1 | 153 | 0.6 | 4.49 | 0.11 to 28.17 | 0.208 |
| **All affected** | 1252 | 5 | 1247 | 0.4 | 2.76 | 0.81 to 7.48 | 0.051 |
| **PSP** | 732 | 3 | 729 | 0.4 | 2.83 | 0.54 to 9.45 | 0.106 |
| **CBS** | 260 | 1 | 259 | 0.4 | 2.66 | 0.06 to 16.55 | 0.324 |
| **APS** | 106 | 0 | 106 | 0.0 | - | - | - |
|  |  | **≥24 Repeats** | **<24 Repeats** | **% ≥24 Repeats** | **Odds ratio** | **95% CI** | **p-value^a^** |
| **Controls** | 15154 | 30 | 15124 | 0.2 | - | - | - |
| **CBD** | 154 | 1 | 153 | 0.6 | 3.29 | 0.08 to 20.09 | 0.269 |
| **All affected** | 1252 | 5 | 1247 | 0.4 | 2.02 | 0.61 to 5.27 | 0.187 |
| **PSP** | 732 | 3 | 729 | 0.4 | 2.07 | 0.40 to 6.69 | 0.193 |
| **CBS** | 260 | 1 | 259 | 0.4 | 1.95 | 0.05 to 11.80 | 0.41 |
| **APS** | 106 | 0 | 106 | 0.0 | - | - | - |
|  |  | **≥23 Repeats** | **<23 Repeats** | **% ≥23 Repeats** | **Odds ratio** | **95% CI** | **p-value^a^** |
| **Controls** | 15154 | 40 | 15114 | 0.3 | - | - | - |
| **CBD** | 154 | 1 | 153 | 0.6 | 2.45 | 0.06 to 14.78 | 0.34 |
| **All affected** | 1252 | 5 | 1247 | 0.4 | 1.51 | 0.47 to 3.85 | 0.39 |
| **PSP** | 732 | 3 | 729 | 0.4 | 1.55 | 0.31 to 4.90 | 0.449 |
| **CBS** | 260 | 1 | 259 | 0.4 | 1.46 | 0.04 to 8.68 | 0.503 |
| **APS** | 106 | 0 | 106 | 0.0 | - | - | - |
|  |  | **≥22 Repeats** | **<22 Repeats** | **% ≥22 Repeats** | **Odds ratio** | **95% CI** | **p-value^a^** |
| **Controls** | 15154 | 46 | 15108 | 0.3 | - | - | - |
| **CBD** | 154 | 1 | 153 | 0.6 | 2.15 | 0.05 to 12.75 | 0.379 |
| **All affected** | 1252 | 5 | 1247 | 0.4 | 1.32 | 0.41 to 3.31 | 0.591 |
| **PSP** | 732 | 3 | 729 | 0.4 | 1.35 | 0.27 to 4.22 | 0.493 |
| **CBS** | 260 | 1 | 259 | 0.4 | 1.27 | 0.03 to 7.49 | 0.551 |
| **APS** | 106 | 0 | 106 | 0.0 | - | - | - |
|  |  | **≥21 Repeats** | **<21 Repeats** | **% ≥21 Repeats** | **Odds ratio** | **95% CI** | **p-value^a^** |
| **Controls** | 15154 | 54 | 15100 | 0.4 | - | - | - |
| **CBD** | 154 | 1 | 153 | 0.6 | 1.83 | 0.05 to 10.77 | 0.427 |
| **All affected** | 1252 | 5 | 1247 | 0.4 | 1.21 | 0.35 to 2.79 | 0.804 |
| **PSP** | 732 | 3 | 729 | 0.4 | 1.15 | 0.23 to 3.56 | 0.747 |
| **CBS** | 260 | 1 | 259 | 0.4 | 1.08 | 0.02 to 6.33 | 0.608 |
| **APS** | 106 | 0 | 106 | 0.0 | - | - | - |
|  |  | **≥20 Repeats** | **<20 Repeats** | **% ≥20 Repeats** | **Odds ratio** | **95% CI** | **p-value^a^** |
| **Controls** | 15154 | 73 | 15081 | 0.5 | - | - | - |
| **CBD** | 154 | 1 | 153 | 0.6 | 1.35 | 0.03 to 7.88 | 0.528 |
| **All affected** | 1252 | 7 | 1245 | 0.6 | 1.16 | 0.45 to 2.52 | 0.671 |
| **PSP** | 732 | 5 | 727 | 0.7 | 1.42 | 0.45 to 3.48 | 0.41 |
| **CBS** | 260 | 1 | 259 | 0.4 | 0.8 | 0.02 to 4.63 | 1 |
| **APS** | 106 | 0 | 106 | 0.0 | - | - | - |
|  |  | **≥19 Repeats** | **<19 Repeats** | **% ≥19 Repeats** | **Odds ratio** | **95% CI** | **p-value^a^** |
| **Controls** | 15154 | 93 | 15061 | 0.6 | - | - | - |
| **CBD** | 154 | 1 | 153 | 0.6 | 1.06 | 0.03 to 6.13 | 0.615 |
| **All affected** | 1252 | 7 | 1245 | 0.6 | 0.91 | 0.36 to 1.96 | 1 |
| **PSP** | 732 | 5 | 727 | 0.7 | 1.11 | 0.35 to 2.71 | 0.806 |
| **CBS** | 260 | 1 | 259 | 0.4 | 0.63 | 0.02 to 3.60 | 1 |
| **APS** | 106 | 0 | 106 | 0.0 | - | - | - |
|  |  | **≥18 Repeats** | **<18 Repeats** | **% ≥18 Repeats** | **Odds ratio** | **95% CI** | **p-value^a^** |
| **Controls** | 15154 | 129 | 15025 | 0.9 | - | - | - |
| **CBD** | 154 | 1 | 153 | 0.6 | 0.76 | 0.02 to 4.38 | 1 |
| **All affected** | 1252 | 7 | 1245 | 0.6 | 0.65 | 0.26 to 1.39 | 0.331 |
| **PSP** | 732 | 5 | 727 | 0.7 | 0.8 | 0.25 to 1.92 | 0.835 |
| **CBS** | 260 | 1 | 259 | 0.4 | 0.45 | 0.01 to 2.57 | 0.729 |
| **APS** | 106 | 0 | 106 | 0.0 | - | - | - |
|  |  | **≥16 Repeats** | **<16 Repeats** | **% ≥16 Repeats** | **Odds ratio** | **95% CI** | **p-value^a^** |
| **Controls** | 15154 | 218 | 14936 | 1.4 | - | - | - |
| **CBD** | 154 | 2 | 152 | 1.3 | 0.90 | 0.11 to 3.36 | 1 |
| **All affected** | 1252 | 13 | 1239 | 1 | 0.72 | 0.38 to 1.26 | 0.317 |
| **PSP** | 732 | 9 | 723 | 1.2 | 0.85 | 0.38 to 1.66 | 0.751 |
| **CBS** | 260 | 2 | 258 | 0.8 | 0.53 | 0.06 to 1.96 | 0.531 |
| **APS** | 106 | 0 | 106 | 0.0 | - | - | - |
|  |  | **≥15 Repeats** | **<15 Repeats** | **% ≥15 Repeats** | **Odds ratio** | **95% CI** | **p-value^a^** |
| **Controls** | 15154 | 302 | 14852 | 2.0 | - | - | - |
| **CBD** | 154 | 2 | 152 | 1.3 | 0.65 | 0.08 to 2.40 | 0.773 |
| **All affected** | 1252 | 18 | 1234 | 1.4 | 0.72 | 0.42 to 1.16 | 0.201 |
| **PSP** | 732 | 13 | 719 | 1.8 | 0.90 | 0.47 to 1.55 | 0.786 |
| **CBS** | 260 | 2 | 258 | 0.8 | 0.38 | 0.05 to 1.40 | 0.253 |
| **APS** | 106 | 1 | 105 | 0.9 | 0.47 | 0.01 to 2.69 | 0.727 |
|  |  | **≥14 Repeats** | **<14 Repeats** | **% ≥14 Repeats** | **Odds ratio** | **95% CI** | **p-value^a^** |
| **Controls** | 15154 | 396 | 14758 | 2.6 | - | - | - |
| **CBD** | 154 | 3 | 151 | 1.9 | 0.74 | 0.15 to 2.22 | 0.801 |
| **All affected** | 1252 | 27 | 424 | 2.2 | 0.82 | 0.53 to 1.23 | 0.403 |
| **PSP** | 732 | 19 | 713 | 2.6 | 0.99 | 0.59 to 1.58 | 1 |
| **CBS** | 260 | 4 | 256 | 1.5 | 0.58 | 0.16 to 1.52 | 0.426 |
| **APS** | 106 | 1 | 105 | 0.9 | 0.35 | 0.01 to 2.03 | 0.531 |
|  |  | **≥13 Repeats** | **<13 Repeats** | **% ≥13 Repeats** | **Odds ratio** | **95% CI** | **p-value^a^** |
| **Controls** | 15154 | 527 | 14627 | 3.5 | - | - | - |
| **CBD** | 154 | 4 | 150 | 2.6 | 0.74 | 0.20 to 1.95 | 0.823 |
| **All affected** | 1252 | 35 | 1217 | 2.8 | 0.80 | 0.55 to 1.13 | 0.225 |
| **PSP** | 732 | 25 | 707 | 3.4 | 0.98 | 0.62 to 1.48 | 1 |
| **CBS** | 260 | 4 | 256 | 1.5 | 0.43 | 0.12 to 1.13 | 0.119 |
| **APS** | 106 | 2 | 104 | 1.9 | 0.53 | 0.06 to 1.99 | 0.590 |
|  |  | **≥12 Repeats** | **<12 Repeats** | **% ≥12 Repeats** | **Odds ratio** | **95% CI** | **p-value^a^** |
| **Controls** | 15154 | 709 | 14445 | 4.7 | - | - | - |
| **CBD** | 154 | 4 | 150 | 2.6 | 0.54 | 0.15 to 1.43 | 0.332 |
| **All affected** | 1252 | 43 | 1209 | 3.4 | 0.72 | 0.52 to 0.99 | 0.041 |
| **PSP** | 732 | 29 | 703 | 4 | 0.84 | 0.55 to 1.23 | 0.418 |
| **CBS** | 260 | 8 | 252 | 3.1 | 0.65 | 0.28 to 1.30 | 0.296 |
| **APS** | 106 | 2 | 104 | 1.9 | 0.39 | 0.05 to 1.46 | 0.244 |
|  |  | **≥11 Repeats** | **<11 Repeats** | **% ≥11 Repeats** | **Odds ratio** | **95% CI** | **p-value^a^** |
| **Controls** | 15154 | 970 | 14187 | 6.4 | - | - | - |
| **CBD** | 154 | 7 | 147 | 4.5 | 0.70 | 0.27 to 1.48 | 0.505 |
| **All affected** | 1252 | 57 | 1195 | 4.6 | 0.70 | 0.52 to 0.92 | 0.009 |
| **PSP** | 732 | 38 | 694 | 5.2 | 0.80 | 0.56 to 1.12 | 0.214 |
| **CBS** | 260 | 9 | 251 | 3.5 | 0.52 | 0.24 to 1.02 | 0.054 |
| **APS** | 106 | 3 | 103 | 2.8 | 0.43 | 0.09 to 1.28 | 0.162 |
|  |  | **≥10 Repeats** | **<10 Repeats** | **% ≥10 Repeats** | **Odds ratio** | **95% CI** | **p-value^a^** |
| **Controls** | 15154 | 1424 | 13730 | 9.4 | - | - | - |
| **CBD** | 154 | 14 | 140 | 9.1 | 0.96 | 0.51 - 1.68 | 1 |
| **All affected** | 1252 | 92 | 1160 | 7.3 | 0.76 | 0.61 to 0.95 | 0.015 |
| **PSP** | 732 | 58 | 674 | 7.9 | 0.83 | 0.62 - 1.09 | 0.193 |
| **CBS** | 260 | 12 | 248 | 4.6 | 0.47 | 0.24 - 0.83 | 0.007 |
| **APS** | 106 | 8 | 98 | 7.5 | 0.79 | 0.33 to 1.62 | 0.618 |
|  |  | **≥9 Repeats** | **<9 Repeats** | **% ≥9 Repeats** | **Odds ratio** | **95% CI** | **p-value^a^** |
| **Controls** | 15154 | 1513 | 13641 | 10.0 | - | - | - |
| **CBD** | 154 | 14 | 140 | 9.1 | 0.90 | 0.48 to 1.57 | 0.892 |
| **All affected** | 1252 | 98 | 1154 | 7.8 | 0.77 | 0.61 to 0.95 | 0.013 |
| **PSP** | 732 | 62 | 670 | 8.5 | 0.83 | 0.63 to 1.09 | 0.205 |
| **CBS** | 260 | 14 | 246 | 5.4 | 0.51 | 0.28 to 0.88 | 0.012 |
| **APS** | 106 | 8 | 98 | 7.5 | 0.74 | 0.31 to 1.51 | 0.515 |
|  |  | **≥8 Repeats** | **<8 Repeats** | **% ≥8 Repeats** | **Odds ratio** | **95% CI** | **p-value^a^** |
| **Controls** | 15154 | 3332 | 11822 | 22.0 | - | - | - |
| **CBD** | 154 | 25 | 129 | 16.2 | 0.69 | 0.43 to 1.07 | 0.096 |
| **All affected** | 1252 | 200 | 1052 | 16 | 0.67 | 0.57 to 0.79 | <0.001 |
| **PSP** | 732 | 119 | 613 | 16.3 | 0.69 | 0.56 to 0.85 | <0.001 |
| **CBS** | 260 | 44 | 216 | 16.9 | 0.72 | 0.51 to 1.00 | 0.049 |
| **APS** | 106 | 12 | 94 | 11.3 | 0.45 | 0.23 to 0.83 | 0.006 |
|  |  | **≥7 Repeats** | **<7 Repeats** | **% ≥7 Repeats** | **Odds ratio** | **95% CI** | **p-value^a^** |
| **Controls** | 15154 | 3560 | 11594 | 23.5 | - | - | - |
| **CBD** | 154 | 25 | 129 | 16.2 | 0.63 | 0.39 to 0.98 | 0.035 |
| **All affected** | 1252 | 220 | 1032 | 17.6 | 0.70 | 0.59 to 0.80 | <0.001 |
| **PSP** | 732 | 135 | 597 | 18.4 | 0.74 | 0.60 to 0.89 | 0.001 |
| **CBS** | 260 | 47 | 213 | 18.1 | 0.72 | 0.51 to 0.99 | 0.046 |
| **APS** | 106 | 13 | 93 | 12.3 | 0.46 | 0.23 to 0.82 | 0.005 |
|  |  | **≥6 Repeats** | **<6 Repeats** | **% ≥6 Repeats** | **Odds ratio** | **95% CI** | **p-value^a^** |
| **Controls** | 15154 | 4505 | 10649 | 29.7 | - | - | - |
| **CBD** | 154 | 30 | 124 | 19.5 | 0.57 | 0.37 to 0.86 | 0.006 |
| **All affected** | 1252 | 293 | 959 | 23.4 | 0.72 | 0.63 to 0.83 | <0.001 |
| **PSP** | 732 | 178 | 554 | 24.3 | 0.76 | 0.64 to 0.90 | 0.002 |
| **CBS** | 260 | 64 | 196 | 24.6 | 0.77 | 0.57 to 1.03 | 0.075 |
| **APS** | 106 | 21 | 85 | 19.8 | 0.58 | 0.34 to 0.95 | 0.025 |
|  |  | **≥5 Repeats** | **<5 Repeats** | **% ≥5 Repeats** | **Odds ratio** | **95% CI** | **p-value^a^** |
| **Controls** | 15154 | 6572 | 8582 | 43.4 | - | - | - |
| **CBD** | 154 | 52 | 102 | 33.8 | 0.67 | 0.47 to 0.94 | 0.018 |
| **All affected** | 1252 | 460 | 792 | 36.7 | 0.76 | 0.67 to 0.86 | <0.001 |
| **PSP** | 732 | 280 | 452 | 38.3 | 0.81 | 0.69 to 0.94 | 0.007 |
| **CBS** | 260 | 92 | 168 | 35.4 | 0.72 | 0.55 to 0.93 | 0.011 |
| **APS** | 106 | 36 | 70 | 34 | 0.67 | 0.44 to 1.02 | 0.061 |
|  |  | **≥4 Repeats** | **<4 Repeats** | **% ≥4 Repeats** | **Odds ratio** | **95% CI** | **p-value^a^** |
| **Controls** | 15154 | 6933 | 8221 | 45.8 | - | - | - |
| **CBD** | 154 | 52 | 102 | 33.8 | 0.60 | 0.42 to 0.85 | 0.003 |
| **All affected** | 1252 | 460 | 792 | 36.7 | 0.69 | 0.61 to 0.78 | <0.001 |
| **PSP** | 732 | 280 | 452 | 38.3 | 0.73 | 0.63 to 0.86 | <0.001 |
| **CBS** | 260 | 92 | 168 | 35.4 | 0.65 | 0.50 to 0.84 | 0.001 |
| **APS** | 106 | 36 | 70 | 34 | 0.61 | 0.40 to 0.93 | 0.018 |

^a^ Bonferroni correction for multiple comparisons applied for 25 intermediate allele cutoffs, giving significance level p = 0.002
