# Supplementary material for: Analysis of C9orf72 repeat length in progressive supranuclear palsy, corticobasal syndrome, corticobasal degeneration, and atypical parkinsonism
Source: J Neurol. 2025 Mar 26;272(4):293. doi: 10.1007/s00415-025-12990-9 (PMC11947049; doi:10.1007/s00415-025-12990-9)
Supplement: Supplementary file 4 — Supplementary file4 (DOCX 21 kb) [file 415_2025_12990_MOESM4_ESM.docx]

**Analysis of *C9orf72* repeat length in progressive supranuclear palsy, corticobasal syndrome, corticobasal degeneration and atypical parkinsonism**

**Journal of Neurology**

**Author List:** David P Vaughan (1,2), Raquel Real (1,2), Marte Theilmann Jensen (1,2), Riona G Fumi (1,2), Megan Hodgson (1,2), Edwin Jabbari (1,2), Danielle Lux (1,2), Lesley Wu (1,2), PROSPECT consortium, MD-GAP, Tom Warner (1,2), Zane Jaunmuktane (2,3), Tamas Revesz (3, 4), James B Rowe (4), Jonathan Rohrer (5), Huw R Morris (1,2)

**Corresponding author:** Huw Morris (h.morris@ucl.ac.uk)- Department of Clinical and Movement Neurosciences, UCL Queen Square Institute of Neurology, University College London, London, UK

**Supplementary Table 3**

**Supplementary Table 3: Cox proportionate hazard modelling survival with various binary cut-offs for repeat size**

| **Diagnosis** | **All** | | **PSP** | | **CBS** | | **APS** | |  | |
| --- | --- | --- | --- | --- | --- | --- | --- | --- | --- | --- |
|  | **HR (95% CI)** | **p-value** | **HR (95% CI)** | **p-value** | **HR (95% CI)** | **p-value** | **HR (95% CI)** |  | **HR (95% CI)** | **p-value** |
| Survival HR, cutoff 30 repeats | 1.21 (0.83 to 0.30) | 0.787^a^ | 3.082e^-7^ (0.00 to Inf) | 0.989^b^ | - | - | - | - | - | - |
| Survival HR, cutoff 29 repeats | 1.8 (0.58 to 5.6) | 0.313^a^ | - | - | 1.412e-07 (0.00 to Inf) | 0.996^c^ | - | - | - | - |
| Survival HR, cutoff 20 repeats | 1.55 (0.58 to 4.15) | 0.385^a^ | 0.56 (0.14 to 2.26) | 0.416^b^ | ^-^ | - | - | - | - | - |
| Survival HR, cutoff 16 repeats | 1.79 (0.85 to 3.80) | 0.126^a^ | 0.73 (0.27 to 1.97) | 0.500^b^ | 1.284e-07 (0.00 to inf) | 0.995^c^ |  | - | - | - |
| Survival HR, cutoff 15 repeats | 1.36 (0.73 to 2.54) | 0.342^a^ | 1.24 (0.58 to 2.63) | 0.578^b^ | ^-^ | - | 1.065e-07 (0.00 to inf) | 0.997^d^ | - | - |
| Survival HR, cutoff 14 repeats | 1.39 (0.80 to 2.41) | 0.244^a^ | 1.26 (0.65 to 2.45) | 0.501^b^ | 0.40 (0.06 to 2.91)^c^ | 0.368^c^ | ^-^ | - | - | - |
| Survival HR, cutoff 13 repeats | 1.18 (0.73 to 1.92) | 0.497^a^ | 1.34 (0.75 to 2.39) | 0.329^b^ | ^-^ | - | 3.641e-08 (0.00 to inf) | 0.998^d^ | 7.6 (0.93 to 62.04) | 0.058^e^ |
| Survival HR, cutoff 12 repeats | 1.17 (0.73 to 1.88) | 0.512^a^ | 1.35 (0.77 to 2.37) | 0.289^b^ | 0.73 (0.18 to 2.98) | 0.656^c^ | ^-^ | - | ^-^ | - |
| Survival HR, cutoff 11 repeats | 1.25 (0.85 to 1.84) | 0.261^a^ | 1.06 (0.66 to 1.70) | 0.812^b^ | 0.96 (0.299 to 3.05) | 0.938^c^ | 1.10 (0.14 to 8.45) | 0.925^d^ | 1.07 (0.37 to 3.05) | 0.904^e^ |
| Survival HR, cutoff 10 repeats | 1.23 (0.91 to 1.67) | 0.179^a^ | 0.90 (0.62 to 1.32) | 0.587^b^ | 0.98 ( 0.39 to 2.42) | 0.959^c^ | 0.47 (0.06 to 3.49) | 0.461^d^ | 0.83 (0.36 to 1.91) | 0.661^e^ |
| Survival HR, cutoff 9 repeats | 1.25 (0.93 to 1.68) | 0.14^a^ | 0.96 (0.67 to 1.38) | 0.813^b^ | 0.80 (0.32 to 1.97) | 0.62^c^ | ^-^ | - | ^-^ | - |
| Survival HR, cutoff 8 repeats | 1.23 (0.99 to 1.52) | 0.063^a^ | 1.14 (0.88 to 1.48) | 0.330^b^ | 0.71 (0.40 to 1.28) | 0.257^c^ | 0.96 (0.29 to 3.24)^c^ | 0.952^d^ | 0.87 (0.46 to 1.66) | 0.680^e^ |
| Survival HR, cutoff 7 repeats | 1.25 (1.02 to 1.54) | 0.033^a^ | 1.09 (0.85 to 1.40) | 0.509^b^ | 0.57 (0.32 to 1.02) | 0.060^c^ | 0.70 (0.21 to 2.31) | 0.558^d^ | ^-^ | - |
| Survival HR, cutoff 6 repeats | 1.22 (1.01 to 1.47) | 0.040^a^ | 1.03 (0.82 to 1.30) | 0.778^b^ | 0.49 (0.30 to 0.82) | 0.007^c^ | 0.48 (0.14 to 4.58) | 0.223^d^ | 0.95 (0.51 to 1.77) | 0.868^e^ |
| Survival HR, cutoff 5 repeats | 1.20 (1.01 to 1.41) | 0.033^a^ | 0.90 (0.73 to 1.11) | 0.335^b^ | 0.62 (0.40 to 0.96) | 0.034c | 1.10 (0.54 to 2.25) | 0.787^d^ | 1.44 (0.88 to 2.35) | 0.144^e^ |
| Survival HR, cutoff 3 repeats | 1.21 (1.021 to 1.42) | 0.023^a^ | 0.85 (0.69 to 1.04) | 0.11^b^ | 0.67 (0.44 to 1.01) | 0.058^c^ | 1.06 (0.52 to 2.16) | 0.881^d^ | 1.47 (0.91 to 2.39) | 0.117^e^ |

Cox proportional hazard regression for survival (sex and age at death or censoring as covariates).

^a^ Bonferroni correction for 16 comparisons, significance level at p = 0.003

^b^ Bonferroni correction for 15 comparisons, significance level at p = 0.003

^c^ Bonferroni correction for 12 comparisons, significance level at p = 0.004

^d^ Bonferroni correction for 9 comparisons, significance level at p = 0.005

^e^ Bonferroni correction for 9 comparisons, significance level at p = 0.007
