# Supplementary material for: Analysis of C9orf72 repeat length in progressive supranuclear palsy, corticobasal syndrome, corticobasal degeneration, and atypical parkinsonism
Source: J Neurol. 2025 Mar 26;272(4):293. doi: 10.1007/s00415-025-12990-9 (PMC11947049; doi:10.1007/s00415-025-12990-9)
Supplement: Supplementary file 5 — Supplementary file5 (PDF 90 kb) [file 415_2025_12990_MOESM5_ESM.pdf]

## PROSPECT Consortium

Khaled Amar (1), Neil Archibald (2), Oliver Bandmann PhD (3), Erica Capps MBChB (4), Alistair Church, MRCGP (5), Jan Coebergh (6), Alyssa Costantini MSc (7), Peter Critchley (8), Boyd CP Ghosh MRCP (9), Michele T.M. Hu, FRCP, PhD (10), Edwin Jabbari, MRCP (7), Christopher Kobylecki, FRCP, PhD (11), P. Nigel Leigh, FRCP, PhD (12), Carl Mann (13), Luke A Massey MRCP (14), Huw R Morris FRCP PhD (7), Uma NathMD FRCP, (15), Nicola Pavese (16), Dominic Paviour, FRCP, PhD (17), James B. Rowe, FRCP, PhD (18), Jagdish Sharma (19), Jenny Vaughan (20,✻)

(1) The Royal Bournemouth Hospital, Department of Medicine and Geriatrics, Bournemouth, UK
(2) The James Cook University Hospital, Marton Road, Middlesbrough, South Tees Hospitals NHS Trust
(3) Sheffield Institute for Translational Neuroscience (SITraN), University of Sheffield, Sheffield, UK
(4) Care of the Elderly Department, Shrewsbury and Telford Hospital NHS Trust, Shrewsbury, UK
(5) Department of Neurology, Royal Gwent Hospital, Newport, UK
(6) Ashford and St Peters NHS Foundation Trust, Chertsey, UK
(7) Department of Clinical and Movement Neurosciences, UCL Queen Square Institute of Neurology, London, UK and Movement Disorders Centre, UCL Queen Square Institute of Neurology, London, UK
(8) University Hospitals Leicester NHS Trust, Department of Neurology
(9) Wessex Neurological Centre, University Hospitals Southampton NHS Foundation Trust, Southampton, UK
(10) Department of Clinical Neurosciences, University of Oxford, UK
(11) Department of Neurology, Salford Royal NHS Foundation Trust, Manchester Academic Health Science Centre, University of Manchester, Manchester, UK
(12) Brighton
(13) University Hospital of the North Midlands
(14) Department of Neurology, Poole Hospital NHS Foundation Trust, Poole, UK
(15) Department of Neurology Sunderland Royal Hospital, Sunderland, UK
(16) Clinical Ageing Research Unit., Newcastle University, Newcastle, UK
(17) Department of Neuroscience, Brighton and Sussex Medical School, Brighton, UK
(18) Department of Clinical Neurosciences and MRC Cognition and Brain Science Unit, University of Cambridge, Cambridge, UK
(19) United Lincolnshire Hospitals NHS Trust
(20) London North West University Healthcare NHS Trust

✻ - deceased

**MDGAP Consortium**

Cornelis Blauwendraat (1,2), Steve Gentleman (3), Djordje Gveric (3), Glenda Halliday (4), John Hardy (5), Andrew King (6), Zane Jaunmuktane (7,8), Tammaryn Lashley (5), Seth Love (9), Christopher Morris (10), Huw R Morris (7) Laura Parkkinen (11), Raquel Real (7) Andrew Robinson (6), Federico Roncaroli (3), Andrew B Singleton (1,2), Colin Smith (12), Claire Shepherd (4), Claire Troakes (6), Thomas T Warner (7,8), Nicholas Wood (7)

(1) Laboratory of Neurogenetics, National Institute on Aging, National Institutes of Health, Bethesda, MD, USA.
(2) Center for Alzheimer's and Related Dementias, National Institute on Aging and National Institute of Neurological Disorders and Stroke, National Institutes of Health, Bethesda, MD, USA.
(3) Department of Brain Sciences, Faculty of Medicine, Imperial College London, London, UK

(4) School of Medical Sciences, Faculty of Medicine and Health, University of Sydney, Sydney, Australia
(5) Department of Neurodegenerative Disease, UCL Queen Square Institute of Neurology, London, UK
(6) Department of Neuroscience, King's College London, London, UK
(7) Department of Clinical and Movement Neurosciences, UCL Queen Square Institute of Neurology, London, UK
(8) Queen Square Brain Bank for Neurological Disorders, UCL Queen Square Institute of Neurology, London, UK
(9) University of Bristol, Bristol, Horfield, United Kingdom
(10) Newcastle Brain Tissue Resource, Institute of Neuroscience, Newcastle University, Newcastle-upon-Tyne, UK
(11) Department of Neuropathology and The Queen's College, University of Oxford, Oxford, UK
(12) Academic Department of Neuropathology, Centre for Clinical Brain Sciences, University of Edinburgh, Edinburgh, UK
(13) Manchester Brain Bank, University of Manchester, Salford, UK
(14) London Neurodegenerative Diseases Brain Bank, King's College London, London, UK
